# Yamaguchi Syndrome: A Hidden Masquerader of Ischemic Heart Disease

**DOI:** 10.7759/cureus.26439

**Published:** 2022-06-29

**Authors:** Anamika Giri, Sourya Acharya, Sandeep Kamat, Samarth Shukla, Sunil Kumar

**Affiliations:** 1 Department of Medicine, Jawaharlal Nehru Medical College, Datta Meghe Institute of Medical Sciences (Deemed to be University), Wardha, IND; 2 Department of Cardiology, Topiwala National Medical College and Bai Yamunabai Laxman (BYL) Nair Charitable Hospital, Mumbai, IND; 3 Department of Pathology, Jawaharlal Nehru Medical College, Datta Meghe Institute of Medical Sciences (Deemed to be University), Wardha, IND

**Keywords:** clinician, left ventricle, prognosis, acute coronary syndrome, hypertrophic cardiomyopathy

## Abstract

Yamaguchi syndrome, also known as apical (Ap) hypertrophic cardiomyopathy (HCM), is a variant of cardiomyopathy that affects the apical region of the left ventricle. ApHCM is frequently misdiagnosed or missed because its symptoms are extremely similar to those of acute coronary syndrome. As clinicians are unfamiliar with this disease, diagnosis can be missed or delayed; as a result, this condition is frequently discovered by chance. ApHCM has a favorable long-term prognosis once properly diagnosed. We report a case of a 50-year-old male who was diagnosed with Yamaguchi syndrome incidentally.

## Introduction

Yamaguchi syndrome, frequently known as apical (Ap) hypertrophic cardiomyopathy (HCM), is an infrequent variant of hypertrophic cardiomyopathy. Isolated hypertrophy of the left ventricular apical region rather than the left ventricular septum characterizes this condition. “Apical HCM” is frequently misdiagnosed as an acute coronary syndrome (ACS), resulting in missed or delayed diagnoses [1]. Chest discomfort, palpitation, dyspnea, syncope, and heart failure are a few of the clinical symptoms. Symptomatic therapy is frequently used in the treatment of this disease. The apical form of HCM has a better prognosis with lower complication and mortality rates than other types of HCM [2].

ApHCM is thought to be autosomal dominant, with the majority of mutations occurring in the genes encoding for the sarcomere. In the thick myofilaments of the heart tissue, the predominant gene mutations occur in “myosin-binding protein C (MYBPC3)” and “myosin heavy chain (MYH7).” Myocardial infarction, atrial fibrillation, ventricular fibrillation, embolic events, and/or congestive heart failure are all possible outcomes of this condition.

## Case presentation

A 52-year-old male came to the emergency department with chief complaints of retrosternal chest pain for two hours, which was sudden in onset and gradually progressing. It was non-radiating and was not associated with breathlessness or palpitations. The patient had no other complaints. The patient was a known case of systemic hypertension for two years on Telmisartan tablet 40 mg OD.

On examination, the pulse rate was 90/min, regular, and the blood pressure was 140/80 mm Hg taken in the right arm supine position. Saturation was 99% on breathing ambient air. The jugular venous pressure was normal. Cardiovascular and other systems did not reveal any abnormality.

**Figure 1:**
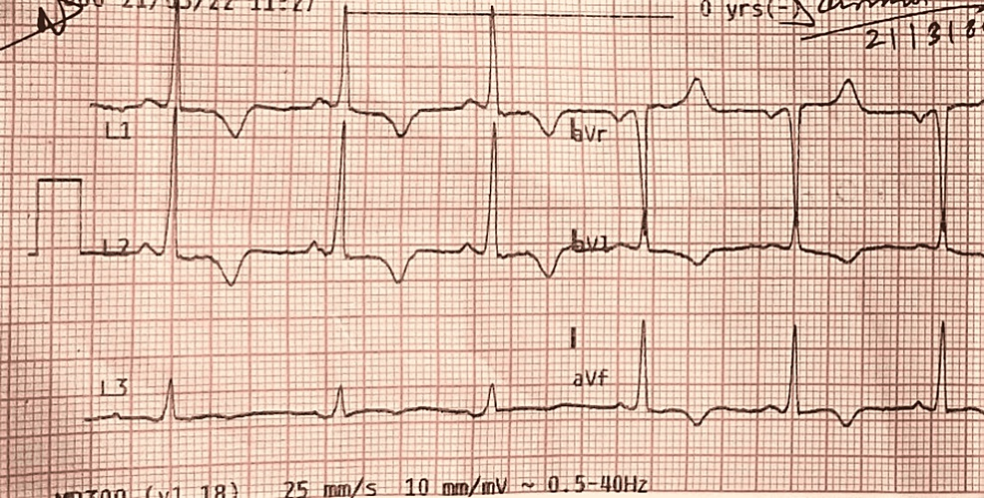
ECG showing deep symmetrical negative “T” waves in limb leads ECG: Electrocardiogram.

**Figure 2:**
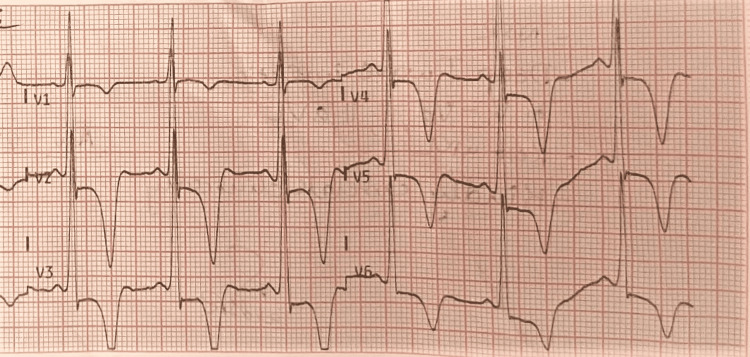
ECG showing deep symmetrical negative “T” waves in precordial leads ECG: Electrocardiogram.

An electrocardiogram (ECG) showed sinus rhythm with left ventricular hypertrophy (LVH) and T wave inversion in the chest as well as the limb lead (Figures 1, 2). Cardiac enzymes and chest x-ray posteroanterior (PA) view were both normal. Left ventricular ejection fraction (LVEF) was 55% on transthoracic echocardiography, along with apical hypertrophy (Video 1). Coronary angiography was done, and it was normal; therefore, the patient was discharged.

**Video 1:** Echocardiography showing obliterative apical left ventricular hypertrophy

## Discussion

Yamaguchi syndrome, frequently referred to as “apical nonobstructive HCM,” is an infrequent variant of HCM in the European population. Sakamoto was the first to report the condition's electrocardiographic pattern and echocardiographic results in Japanese patients in 1976, while Yamaguchi was the first to characterize the syndrome and its ventriculographic features in 1979 [3].

On auscultation, the presence of fourth heart sound (S4), deep negative T waves on the ECG notably in the precordial leads, and a "spade-like" structure of the left ventricular chamber at end-diastole on left ventriculography and also in 2D echo are all common signs of apical HCM. Apical wall motion anomalies like hypokinesis and aneurysm development can be present in patients with apical hypertrophic cardiomyopathy” [4].

ECG findings in a patient of Yamaguchi syndrome are that of LVH and giant (>10 mm in amplitude) negative T waves most prominent in V4-5 chest leads, which may be directly proportional to the severity of apical hypertrophy [5]. Echocardiography, computed tomography, left ventriculography, and most importantly cardiac magnetic resonance imaging (cardiac MRI) can all be used to diagnose ApHCM. The most accurate assessment is obtained via multimodality imaging, which combines many of these imaging techniques. ApHCM is treated in the same way that HCM patients are. ApHCM, on the other hand, is usually associated with modest risk and practically never necessitates the insertion of an implanted cardioverter-defibrillator for primary prevention. These symptoms are quite frequent in middle-aged or elderly patients who may also have this related pathology, ruling out that coronary artery disease is critical in these patients [6]. It is also crucial to figure out what kind of HCM the patient has since apical HCM has a favorable long-term prognosis [7].

## Conclusions

Traditionally, Yamaguchi syndrome was thought to be a disease involving just the Japanese population. Thus, it is of utmost importance that physicians rule out this rare entity as a possible differential diagnosis in patients having symptoms like angina predominantly. It is to be also kept in mind that this can be found in asymptomatic subjects. Initial ECG usually hints at the underlying pathology. So, any ECG which is suggestive of ischemic heart disease may unfold this rare disorder. This will ensure there is not any diagnostic delay and can also help in preventing the progression of the disease to a fatal stage.
